# Automatic Lung Tumor Segmentation on PET/CT Images Using Fuzzy Markov Random Field Model

**DOI:** 10.1155/2014/401201

**Published:** 2014-05-29

**Authors:** Yu Guo, Yuanming Feng, Jian Sun, Ning Zhang, Wang Lin, Yu Sa, Ping Wang

**Affiliations:** ^1^Tianjin Key Lab of BME Measurement, Tianjin University, Tianjin 300072, China; ^2^Department of Radiation Oncology, Tianjin Medical University Cancer Institute and Hospital, Tianjin 300060, China

## Abstract

The combination of positron emission tomography (PET) and CT images provides complementary functional and anatomical information of human tissues and it has been used for better tumor volume definition of lung cancer. This paper proposed a robust method for automatic lung tumor segmentation on PET/CT images. The new method is based on fuzzy Markov random field (MRF) model. The combination of PET and CT image information is achieved by using a proper joint posterior probability distribution of observed features in the fuzzy MRF model which performs better than the commonly used Gaussian joint distribution. In this study, the PET and CT simulation images of 7 non-small cell lung cancer (NSCLC) patients were used to evaluate the proposed method. Tumor segmentations with the proposed method and manual method by an experienced radiation oncologist on the fused images were performed, respectively. Segmentation results obtained with the two methods were similar and Dice's similarity coefficient (DSC) was 0.85 ± 0.013. It has been shown that effective and automatic segmentations can be achieved with this method for lung tumors which locate near other organs with similar intensities in PET and CT images, such as when the tumors extend into chest wall or mediastinum.

## 1. Instruction 

Combination of positron emission tomography (PET) and CT images provides complementary functional and anatomical information which has been used for tumor volume definition in radiation treatment (RT) planning for lung cancer patients [1]. Automatic methods for tumor segmentation on PET/CT images are highly desired to avoid the inter- and intraobserver variability caused by manual method.

Many automatic tumor segmentation techniques for identification and delineation of cancerous tissues have been reported such as for brain tumor [2], lung tumor [3], and prostate tumor [4]. The segmentation can be performed either on a single image set, such as CT [5], PET [6], or MRI images [7], or on the fused image set of different image modalities such as CT/PET [8–10] or multiparametric MRI images [2, 11]. Different types of tumors and image modalities have different image features, and thus different segmentation strategy should be developed for effective and accurate tumor segmentations.

CT images provide anatomical information with high spatial resolution. However, for the lung tumors abutting or involved in adjacent structures such as chest wall, mediastinum, or diaphragm which show intensities similar to those of tumors on the images, it is difficult to distinguish them from the adjacent tissues with commonly used autosegmentation algorithms. Lung tumors can be distinguished from the adjacent tissues on PET images, but the segmentation accuracy is still limited due to the coarser spatial resolution of the image data and motion artifacts as the result of time-consuming procedure of data acquisition. Therefore, one of the key points of lung tumor segmentation on PET/CT images is to combine the advantages of the two image modalities effectively.

Several methods [8–10] are proposed for lung tumor segmentation on PET/CT images. Most of the reported methods fuse different features extracted from PET and CT images inside one single *N*-dimensional vector. In this paper, we propose a new strategy for fusing PET and CT information. The method is based on fuzzy Markov random field (MRF) model which has shown effective performance for unsupervised image segmentation [12, 13]. Different from traditional fuzzy MRF method, the proposed method designs a new joint posterior probabilistic model for effective combination of PET and CT image information. The new method was evaluated using image data of 7 patients with lung cancer in this study and experimental results showed its good performance in automatic tumor delineation.

This paper is organized as follows. We first present the basic theory about image segmentation using fuzzy MRF model in Section 2.1. The framework of lung tumor segmentation on CT/PET images using fuzzy MRF model is then described in Section 2.2. The evaluation of the proposed method and quantification results are shown in Section 3. Finally, discussion and conclusion are presented at the end.

## 2. Materials and Methods

### 2.1. Image Segmentation Based on Fuzzy MRF Model

The fuzzy MRF model is an unsupervised statistical methodology that takes place in Bayesian framework. Image segmentation based on fuzzy MRF model requires modeling two random fields [14]. For the set of pixels *S* = {1,…, *N*} of an image to be segmented, *Y* = (*y*_*s*_)_*s*∈*S*_ is the observed random field which represents the observed image and takes its value in the set of real numbers, while *X* = (*x*_*s*_)_*s*∈*S*_ is the unobserved random field, which corresponds to the final segmentation results and takes its value in the set of {1,2,…, *k*,…, *K*}, with *K* being the number of classes. In comparison to the standard implementation where only a finite number of hard classes are considered, fuzzy segmentations allow each pixel to belong simultaneously to more than one class. From this point of view, *x*_*s*_ which is the realization of the random field *X* for the pixel at location *s* should be associated with a vector [*x*_*s*1_, *x*_*s*2_,…, *x*_*sk*_,…, *x*_*sK*_]^*T*^ with *x*_*s*1_ + *x*_*s*2_ + ⋯+*x*_*sk*_ + ⋯+*x*_*sK*_ = 1, where *x*_*sk*_ is the membership degree of the pixel to class *k*. The segmentation problem consists of estimating **x**_*s*_ = [*x*_*s*1_,…,*x*_*sk*_,…,*x*_*sK*_]^*T*^ (*s* ∈ *S*) from the available noisy observation.

The relationship between *X* and *Y* can be modeled by the joint distribution *P*(*X*, *Y*). According to Bayesian principle, we have *P*(*X*, *Y*) = *P*(*Y* | *X*)*P*(*X*), where *P*(*X*) is the prior distribution of *X* and *P*(*Y* | *X*) is the posterior distribution. In the fuzzy MRF model, *P*(*X*) is assumed to be stationary and Markovian.

Image segmentation problem is considered as a maximum a posteriori (MAP) problem. That is to estimate the membership degree matrix **x** = [**x**_1_; **x**_2_; …; **x**_*N*_]^*T*^ of the studied image which maximizes probability density function (PDF) *p*(**x** | **y**), where *N* is the number of pixels in the image and **y** represents the intensity or feature vector of the image. There is $\overset{\wedge }{x}=\arg\max_{x}p(x|y)$. In Bayesian framework, *p*(**x** | **y**) = *p*(**y** | **x**)*p*(**x**)/*p*(**y**), where *p*(**y**) can be considered independent of *p*(**x**). Therefore, we have

(1) x∧  =argmax⁡x p(y ∣ x)p(x)=argmax⁡x[ln⁡p(y ∣ x)+ln⁡p(x)].

The probabilistic models used for *p*(**y** | **x**) and *p*(**x**) are based on the prior knowledge of the studied image. In fuzzy MRF model, *X* is set as Gibbs distribution for the reason that pixels tend to belong to the same class with their neighbors. For the posterior distribution *p*(**y** | **x**), Gaussian distribution is usually used, since, for a region with common properties in images, it is reasonable to assume that the intensity or other feature values are distributed around the mean value of the class. As long as proper probabilistic models are determined, the membership degree matrix **x** can be estimated by solving the maximization problem in (2).

### 2.2. Tumor Segmentation on PET/CT Images Using Fuzzy MRF Model

In this subsection, the fuzzy MRF method for tumor segmentation on PET/CT images is described in detail. For segmenting tumor on PET/CT images, *x*_*s*_ is the membership degree of the voxel at location *s* to tumor class. *y*_*s*_ is related to some image features of the voxel at location *s* extracted from PET and CT images. Here, the CT image intensity *y*_CT_ and the standardized uptake value (SUV) *y*_SUV_ derived from PET images are used. Therefore, tumor segmentation on PET/CT image using fuzzy MRF model is actually done to estimate the membership degree to tumor class of each voxel by solving the maximization problem as shown in

(2) $$\begin{array}{c} \overset{\wedge }{x}\;\;=\arg\underset{x}{\max}\left[\ln p\left(y_{\text{CT}},y_{\text{SUV}}\;|\;x\right)+\ln p\left(x\right)\right]. \end{array}$$

One of the key points about the above maximization problem is to choose proper probabilistic models for *p*(**y**_CT_, **y**_SUV_ | **x**) and *p*(**x**). Since regions which show tumor features on both PET and CT images may be tumor regions with higher possibility, we set *p*(**y**_CT_, **y**_SUV_ | **x**) as **x**min⁡[*p*_11_(**y**_SUV_), *p*_21_(**y**_CT_)]+(1 − **x**)max⁡[*p*_10_(**y**_SUV_), *p*_20_(**y**_CT_)], where *p*_11_ and *p*_21_ are the PDFs of the two features given that the studied voxels belong to tumor class, while *p*_10_ and *p*_20_ are the PDFs of the two features given that the voxels belong to normal tissue class. *p*_21_ and *p*_20_ are set as Gaussian functions, since the CT intensities of tumor tissues and normal tissues around the tumor tissues are usually assumed to be distributed around the mean value of their own class. Besides, it is reasonable to assume that SUVs of normal tissues have a normal distribution, so *p*_10_ is also set as a Gaussian function. For *p*_11_(**y**_SUV_), a uniform distribution is used, which means that voxels with a SUV value greater than a threshold have the same possibility to be tumor class. The parameters of *p*_11_, *p*_21_, *p*_10_, and *p*_20_ are estimated by fitting the histogram of the region obtained from *C*-means clustering with the selected distributions. The prior distribution of *p*(**x**) is set as Gibbs distribution as in other fuzzy MRF methods [13]. As a result, the final maximization problem can be noted as

(3) $$\begin{array}{c} \overset{\wedge }{x}\;\;=\arg\underset{x}{\max}\;C\left(x\right), \end{array}$$

with

(4) C(x)=∑i{ln⁡[ximin⁡[p11(yiSUV),p21(yiCT)]+(1−xi)max⁡[p10(yiSUV),p20(yiCT)]]   −β∑j∈Ri(xi−xj)2},

where *i* and *j* are the image indexes, *x*_*i*_ is the membership degree of the *i*th voxel belonging to tumor class, *R*_*i*_ is the neighborhood (5 × 5 × 3) of the *i*th voxel, and *β* is the smoothing parameter which affects the smoothness of segmentation results. Besides, *p*_10_(*y*_*i*SUV_) can be computed with the following function:

(5) $$\begin{array}{c} p_{10}\left(y_{i\text{SUV}}\right)=\frac{1}{\sqrt{2\pi \sigma_{10}^{2}}}\exp\left(-\frac{{\left(y_{i\text{SUV}}-\mu_{10}\right)}^{2}}{2\sigma_{10}^{2}}\right), \end{array}$$

where *μ*_10_ and *σ*_10_^2^ are, respectively, the mean and variance of the Gaussian function in (5). The function *p*_11_(*y*_*i*SUV_) is shown as follows;

(6) $$\begin{array}{c} p_{11}\left(y_{i\text{SUV}}\right)=\{\begin{array}{cc} 0, & y_{i\text{SUV}}<a,\\ \frac{1}{b-a}, & y_{i\text{SUV}}\geq a, \end{array} \end{array}$$

where *a* is a SUV threshold to distinguish tumor tissues from normal tissues and *b* is the maximum SUV value of the PET image studied.

Gradient decent algorithm is used to solve the optimization problem in (3). The iterative form of the proposed method can be summarized as follows.

1. Initialize the vector of membership degree **x**(0) = [*x*_1_(0), *x*_2_(0),…, *x*_*N*_(0)].
2. Update **x**(*n* + 1) = **x**(*n*) − *α*Δ**x**(*n*), where Δ**x**(*n*) = [Δ*x*_1_(*n*), Δ*x*_2_(*n*),…, Δ*x*_*N*_(*n*)] and Δ*x*_*i*_(*n*) = ∂[−*C*(**x**(*n*))]/∂*x*_*i*_(*n*).
3. Repeat step (2) until ||**x**(*n*+1)−**x**(*n*)||_2_ ≤ *ε*, where ||·||_2_ represent 2-norm and *ε* is the stopping threshold set as a small positive real number.

In the proposed method, the step size *α* and the smoothing parameter *β* need to be selected. A large step size may make the algorithm divergent, so using a small step size is common practice. *β* should be selected according to the smoothness of tumor tissues on images. Once the membership degree vector **x** is estimated, we determine the final tumor regions using a simple threshold method.

## 3. Experiments and Results

The PET and CT simulation images of 7 non-small cell lung cancer (NSCLC) patients were used to evaluate the proposed method. PET and CT fusion results of studied images using MIM 5.2 (MIM Software) were exported to in-house-developed software for the study. Gross tumor volume (GTV) delineation with the proposed MRF method and manual method by an experienced radiation oncologist on the fused images were performed, respectively. The manually contoured GTVs were checked and confirmed by another experienced radiation oncologist. The robustness of MRF method was evaluated by comparing the overlap of the two delineations using Dice's similarity coefficient (DSC) expressed as 2(*v*1∩*v*2)/(*v*1 + *v*2), where *v*1 is the manually delineated GTV volume and *v*2 is the GTV volume obtained with the new automatic method.

**Figure 1:**
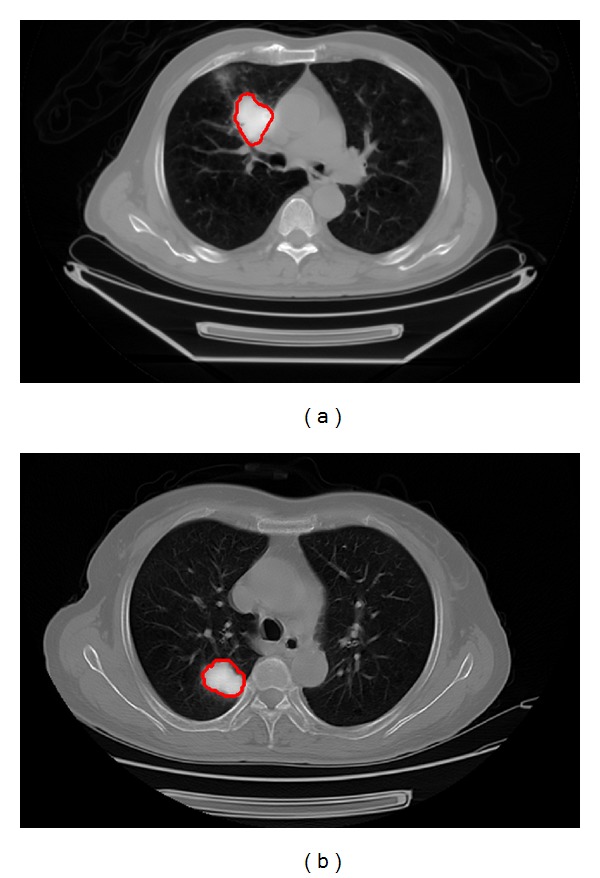
Fused PET/CT images of two patients with lung tumors delineated by radiation oncologist shown in red lines.

Some lung tumors in the studied cases locate near other organs with similar intensities to tumor tissues in PET and CT images (such as the chest wall or the mediastinum), and it is difficult to segment these lung tumors.  Figure 1(a) shows a fused PET/CT image of a patient with lung cancer; the tumor locates close to the mediastinum and has image features similar to those of the mediastinum.  Figure 1(b) shows another case in which the tumor is close to the chest wall.

**Figure 2:**
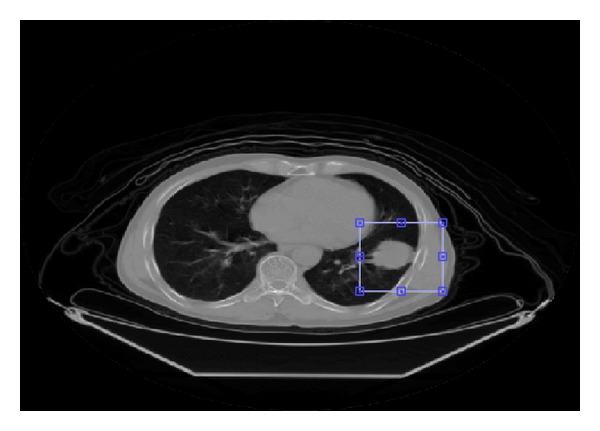
An axial CT slice of a patient with the region to be segmented marked.

**Figure 3:**
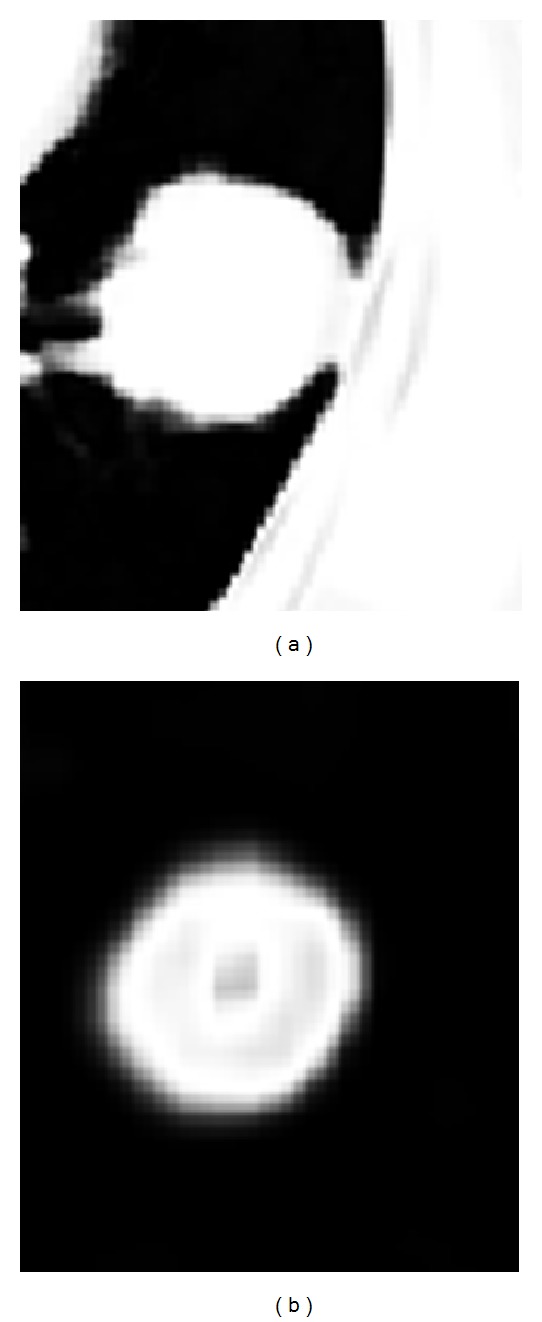
Membership degrees to tumor class of the selected region in Figure 2 obtained with fuzzy *C*-means clustering; (a) only CT intensity feature is used; (b) only SUV feature is used.

**Figure 4:**
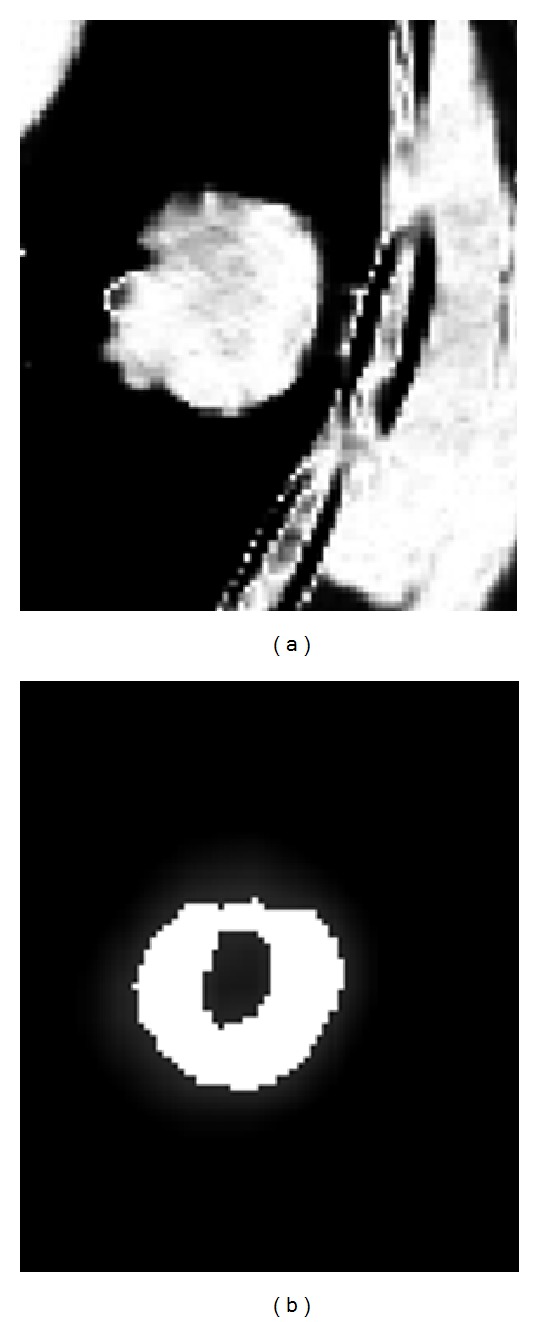
Probabilities of tumor class of the voxels within the selected region in Figure 2, (a) *p*_21_(*y*_CT_); (b) *p*_11_(*y*_SUV_).

The proposed segmentation algorithm performs on a manually selected region rather than the whole volume enclosed by the image set. Lung tumors locate in the selected regions, as shown in Figure 2. There are much fewer tissue components in the selected region than in the whole volume which makes the segmentation easier. In order to obtain the probabilistic model parameters of *p*_10_, *p*_11_, *p*_20_, and *p*_21_ in (3), the fuzzy *C*-means clustering [15] is applied to the regions of interest in the studied PET and CT images.  Figure 3 shows the estimated membership degrees of the selected region in Figure 2 to tumor class with fuzzy *C*-means clustering. It is assumed that the voxels which have the membership degrees greater than 0.1 belong to tumor class. Then, we can have a rough segmentation of the selected region. According to the segmentation result, the desired probabilistic model parameters, such as the means and deviations of Gaussian models in (5), can be estimated.  Figure 4 shows the probabilities of tumor class of the voxels in the selected region of Figure 2, which are computed based on the probabilistic model parameters estimated in the previous step.

**Figure 5:**
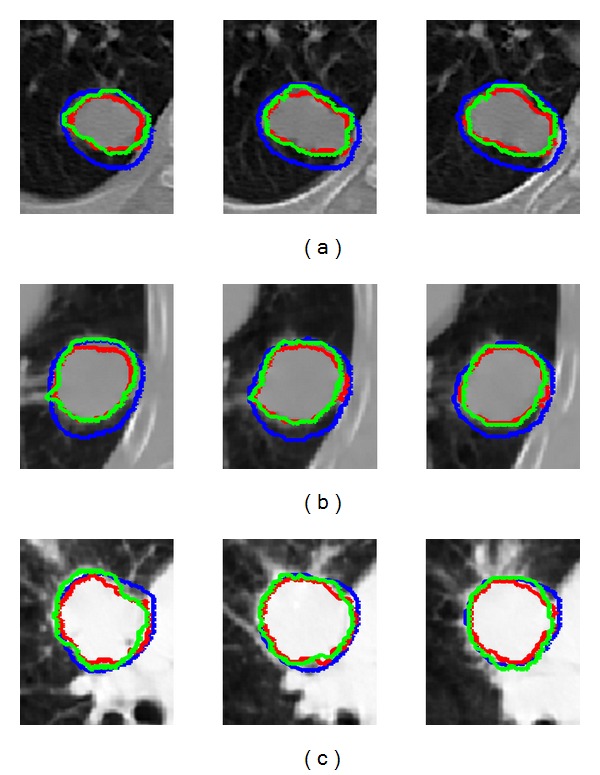
GTVs in axial CT slices of patient (a), (b), and (c). GTVs in blue are the results with fuzzy MRF method using only PET images and GTVs in red and green are the results with the new method and manual method, respectively, using both PET and CT images.

Figure 5 shows the tumor segmentation results of 3 patients obtained, respectively, with the manual method, fuzzy MRF method using only PET images, and the proposed method using CT/PET images. As shown in Figure 5, lung tumors in these cases are close to other tissues with similar image features. We can also see that GTVs determined by using only PET images are bigger than the ones obtained with the other two methods in most cases, while GTVs obtained with the manual method and the proposed method are similar. For the other cases, we obtained the same results.

In our work, the traditional MRF model method in [13] and fuzzy *C*-means clustering are also used to segment lung tumors. CT/PET image based segmentation results obtained with the two methods are much worse than the results of the two methods using only PET images. The DSCs of the two methods for all the studied cases were 0.59 ± 0.034 and 0.62 ± 0.029, respectively. It means that the two methods cannot effectively combine CT and PET image information to achieve accurate lung tumor segmentation. DSC of the proposed method for all the studied cases was 0.85 ± 0.013. Therefore, the proposed method is able to effectively utilize CT and PET image information and achieves good lung tumor segmentation.

## 4. Conclusions

CT/PET images provide complementary functional and anatomical information of human tissues and lead to better lung tumor definition. This paper proposes a fuzzy MRF model based method for automatic lung tumor segmentation on CT/PET images. Different from traditional fuzzy MRF model method, it utilizes a new joint posterior probabilistic model, which can effectively take advantage of both CT and PET image information for the identification and delineation of tumor volume. Experimental results show its good performance. For lung tumors which locate near other tissues with similar intensities in PET and CT images, such as when they extend into the chest wall or the mediastinum, this method was able to achieve more effective tumor segmentation. In future work, we will further test the reliability of this method with more clinical data.
